# Domestic Cat Hepadnavirus Antigens in Lymphoma Tissues. Comment on Beatty et al. Domestic Cat Hepadnavirus and Lymphoma. *Viruses* 2023, *15*, 2294

**DOI:** 10.3390/v16010148

**Published:** 2024-01-19

**Authors:** Chutchai Piewbang, Sabrina Wahyu Wardhani, Jedsada Siripoonsub, Sirintra Sirivisoot, Anudep Rungsipipat, Somporn Techangamsuwan

**Affiliations:** 1 Department of Pathology, Faculty of Veterinary Science, Chulalongkorn University, Bangkok 10330, Thailand; alkaline_eart@hotmail.com (C.P.); sabrina.w.wardhani@gmail.com (S.W.W.); jedsada007@hotmail.com (J.S.); sirintra.s@chula.ac.th (S.S.); anudep.r@chula.ac.th (A.R.); 2 Animal Virome and Diagnostic Development Research Unit, Faculty of Veterinary Science, Chulalongkorn University, Bangkok 10330, Thailand; 3 Center of Excellence for Companion Animal Cancer, Department of Pathology, Faculty of Veterinary Science, Chulalongkorn University, Bangkok 10330, Thailand

We are addressing the comments made by Beatty et al. [1] in response to the commentary on our publication about the detection of domestic cat hepadnavirus (DCH) DNA in cats with lymphoma [2]. DCH, a hepadnavirus initially identified in a cat with lymphoma, is closely related to human hepatitis B virus (HBV). While the identification of DCH in cats has prompted inquiries about feline heath, several studies have established a link between the detection of DCH DNA and changes in liver biochemistry, lymphoplasmacytic hepatitis, and hepatocellular carcinomas [3,4,5]. Although a conclusive link between DCH infection and the aforementioned diseases is still a topic of discussion, there is an indication that DCH infection may contribute to clinical features similar to those described in human HBV infection [6,7]. The growing number of reported extrahepatic manifestations in HBV infection includes an association with an increased risk of non-Hodgkin’s lymphoma (NHL), extensively documented in various human studies [8,9,10,11,12]. Given the relative nature of DCH to HBV and the frequent occurrence of lymphoma in cats [13], exploring the epidemiology of DCH in cats with lymphoma holds the potential to reveal an additional dimension to the impact of DCH on feline health. 

We acknowledge the commentary made by Beatty et al. [1], which specifically raised independent concerned points of non-agreement with our study. While Beatty et al. may not be aware of the potential risk of B-cell lymphoma associated with DCH infection analogous to the connection observed in chronic HBV infection in humans where lymphoma development has not been extensively studied in other species, our study unveiled a heightened probability of detecting DCH DNA in cats with lymphoma. Although there is no existing research on the development of lymphoma in cases of chronic hepadnavirus infection in other susceptible animals, this does not rule out the plausible association of this disease. Reports have proposed various factors including prolonged antigenic stimulation and direct infection in lymph nodes, recognized as potential factors in NHL development in chronic HBV infection [14,15,16,17,18], and these phenomena have also been found in woodchuck hepatitis virus infection [17,19,20,21]. Prolonged DCH viremia, coupled with evidence of DCH localization in the lymph nodes, as indicated in recent studies [22,23,24], suggests the possibility of chronic antigenic stimulation caused by DCH infection. Consequently, we have advocated for additional studies to explore and substantiate our findings. In addressing this disagreement, we emphasize the importance of conducting further scientific investigations rather than resorting to conclusions without additional empirical evidence. 

Concerning the initial point raised by Beatty et al. [1], we concur that the absence of DCH DNA detection does not definitively establish non-infection cases. We posit that a conclusive determination of infection status requires the detection of DCH antibodies in conjunction with the identification of DCH DNA. Unfortunately, due to the unavailability of commercially accessible anti-DCH antibodies, this determination was not feasible within the confines of the present study. It is essential to highlight that we thoroughly addressed this limitation in the original publication [2]. Additionally, we emphasize the need for further investigations to validate our initial observation regarding the prevalence of DCH in lymphoma cases.

Secondly, Beatty et al. [1] expressed reservations about the conclusiveness of our DCH in situ hybridization (ISH) results in lymphoma tissues, raising multiple concerns. Consequently, we have comprehensively addressed their detailed opinions. Beatty et al. raised the point that ISH was conducted in cases with detected DCH DNA in sera. However, it is important to clarify that our ISH was specifically performed on lymphoma sections from cats with DCH qPCR-positive lymphoma tissues [2]. This contrasts with the commentary by Beatty et al., who wrote that ISH was solely conducted on cats with positive DCH DNA in sera.

Thirdly, we wish to emphasize that all lymphoma sections positive for DCH qPCR underwent ISH (see Table 3 and Figure 1 in Piewbang et al. [2]), in contrast to Beatty et al.’s commentary of determination from a single case. We acknowledge the possibility that they might have overlooked or misunderstood this particular set of data. Beatty et al. also indicated their personal observations, expressing uncertainty about the conclusiveness of our illustration, citing issues such as the absence of negative controls and the possibility of staining patterns being non-specific. To address these concerns, it is essential to clarify that we implemented appropriate negative controls for ISH. This involved incubating DCH-positive lymphoma sections with a non-related probe and another set of DCH-positive lymphoma sections with DNAse enzyme before exposure to the DCH probe [2]. The purpose was to degrade all resident DNA, including DCH DNA and any irrelevant DNA that might lead to non-specific binding. Subsequently, our slides were blocked with bovine serum albumin, as detailed in the protocols used in various studies to prevent non-specific protein binding [25]. Therefore, we are confident that our negative controls for ISH sufficiently demonstrate the robustness of our ISH procedures. In light of these precautions, any assertion of non-specific staining, as suggested by Beatty et al., is unlikely.

Certainly, it is crucial to reiterate that, despite the concerns raised by Beatty et al. regarding a potential non-specific staining pattern linked to endogenous alkaline phosphatase activity, our careful validation process contradicts this notion. If endogenous alkaline phosphatase activity were the cause, a broader range of cells in the investigated tissue would likely have displayed hybridization signals. Our stringent procedures, including the use of appropriate negative controls, affirm that the signals observed in our ISH are genuinely indicative of DCH–DNA hybridization. We maintain our confidence in the specificity and accuracy of our ISH results, reinforcing the evidence of DCH presence in the lymphoma sections.

To provide additional support for our discovery of DCH DNA localized in feline lymphoma tissues, we have included an additional illustration of DCH-ISH in lymphoma sections (Figure 1). We performed the ISH following the protocols and DCH probe use as described in Piewbang et al. [2] to repeat the robustness. This is complemented by negative controls in the same field and magnification, clearly demonstrating the specificity of our findings. The hybridization signals were prominently observed in lymphoma tissues, with no reaction observed in the negative control section. Furthermore, we conducted immunohistochemistry (IHC) to detect DCH protein using anti-HBV core antigen (HBcAg), a method that has shown cross-reactivity with DCH core protein in previous studies [4,24] and has been employed to ascertain DCH infection [24,26].

The IHC results consistently support the presence of DCH proteins localized in the lymphoma sections, reinforcing the outcomes of DCH-qPCR and ISH. Positive IHC results also suggest the translation activity of DCH DNA in lymphoma tissues. While our study may not definitively conclude upon an association between DCH infection and lymphoma development, we believe that our findings present unique insights that could potentially serve as evidence for DCH infection impacting feline health. We remain convinced that further investigations are essential to establish DCH infection as a potential risk factor for feline lymphoma, akin to what has been observed in HBV infection. 

Finally, we express sincere appreciation for the insightful commentary on our peer-reviewed study entitled “Domestic cat hepadavirus antigen in lymphoma tissues”. This scientific and evidence-based study undoubtedly enhances the credibility of our findings. We also emphasize that the commentary made by Beatty et al. alone, without additional investigations, may be insufficient and has the potential to be misleading.

## Figures and Tables

**Figure 1:**
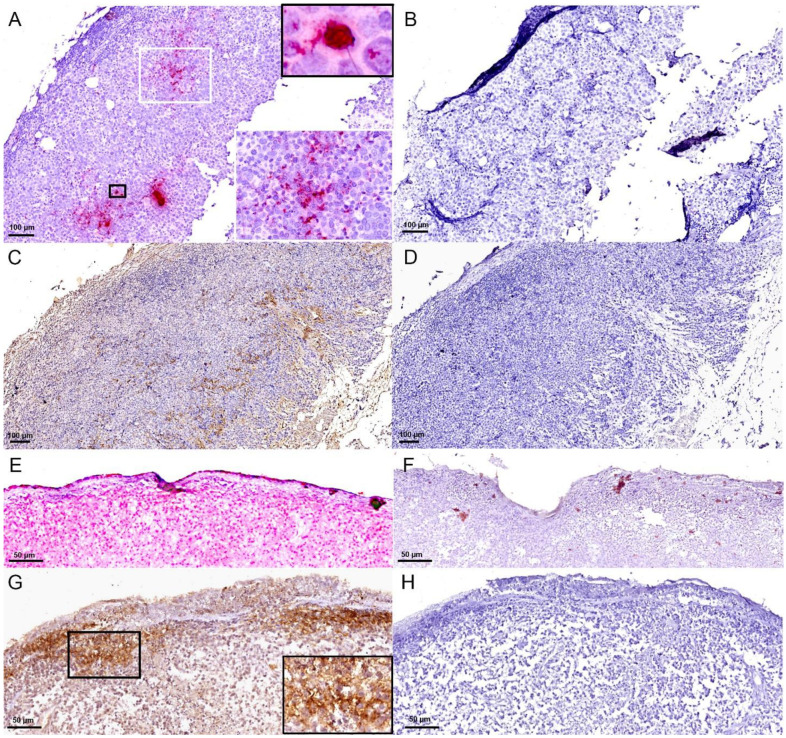
DCH infection in cats with lymphoma. Lymph node: cat no. 3 (**A**–**D**) and cat no. 4 (**E**,**F**). (**A**) Multifocal, strong red dot-like pattern of DCH DNA in the lymphoma section (white inset). Prominent nuclear labeling of DCH DNA (black inset). (**B**) No DCH DNA hybridization presented in the DCH qPCR-positive lymphoma section incubation with feline bocavirus-3 DNA probe. In situ hybridization (ISH) of DCH (**A**,**B**). (**C**) Multifocal immunolabelling (brownish color) of DCH core protein presented in the lymphoma section. No immunoreactivity was present in the DCH qPCR-positive lymphoma section incubation with normal rabbit IgG antibody. Horseradish peroxidase immunohistochemistry (IHC) (**C**,**D**). (**E**) Diffuse, intense hybridization signals in the lymphoma section. (**F**) No specific hybridization reactions were presented in the cellular morphology of the negative control section. DCH ISH (**E**,**F**). (**G**) Multifocal, cytoplasmic labeling (inset) of DCH core protein in the lymphoma section by IHC. (**H**) No labeling was observed in the negative control section. IHC for DCH core protein (**G**,**H**).
